# Exosome purification based on PEG-coated Fe_3_O_4_ nanoparticles

**DOI:** 10.1371/journal.pone.0199438

**Published:** 2018-06-22

**Authors:** Ming Chang, Yaw-Jen Chang, Pei Yu Chao, Qing Yu

**Affiliations:** 1 Key Laboratory of Process Monitoring and System Optimization for Mechanical and Electrical Equipment in Fujian Province, Huaqiao University, Xiamen, China; 2 Department of Mechanical Engineering, Chung Yuan Christian University, Chung Li District, Taoyuan City, Taiwan; Helsingin Yliopisto, FINLAND

## Abstract

Cancer cells secrete many exosomes, which facilitate metastasis and the later growth of cancer. For early cancer diagnosis, the detection of exosomes is a crucial step. Exosomes exist in biological fluid, such as blood, which contains various proteins. It is necessary to remove the proteins in the biological fluid to avoid test interference. This paper presented a novel method for exosome isolation using Fe_3_O_4_ magnetic nanoparticles (MNPs), which were synthesized using the chemical co-precipitation method and then coated with polyethylene glycol (PEG). The experimental results showed that the diameter of the PEG-coated Fe_3_O_4_ nanoparticles was about 20 nm, while an agglomerate of MNPs reached hundreds of nanometers in size. In the protein removal experiments, fetal bovine serum (FBS) was adopted as the analyte for bioassays of exosome purification. PEG-coated Fe_3_O_4_ MNPs reduced the protein concentration in FBS to 39.89% of the original solution. By observing a particle size distribution of 30~200 nm (the size range of various exosomes), the exosome concentrations were kept the same before and after purification. In the gel electrophoresis experiments, the bands of CD63 (~53 kDa) and CD9 (~22 kDa) revealed that exosomes existed in FBS as well as in the purified solution. However, the bands of the serum albumins (~66 kDa) and the various immunoglobulins (around 160 ~ 188 kDa) in the purified solution’s lane explained that most proteins in FBS were removed by PEG-coated Fe_3_O_4_ MNPs. When purifying exosomes from serum, protein removal is critical for further exosome investigation. The proposed technique provides a simple and effective method to remove proteins in the serum using the PEG-coated Fe_3_O_4_ MNPs.

## Introduction

Cancer, also known as malignant tumors, refers to the abnormal proliferation of cells, and these abnormal cells may invade other parts of the body. For many years, cancer has been at the top of the list of the ten main causes of death, and metastasis is the main cause of cancer deaths [1–3]. Recent studies have confirmed that cancer cells, before metastasis, will release exosomes, which facilitate the metastasis and the later growth of cancer [4]. The integrin on the surface of the exosome equips it with organotropism and targets specific cells. These two characteristics can accurately determine the organ destination for the exosomes secreted by cancer cells [5]. Once these products arrive at the distal organ, they work to create an environment that is ideal for cancer growth [6,7]. Based on this logic, if an unusually large number of exosomes are found through a blood test, it could be the precursor of cancer metastasis [8,9]. Therefore, the early discovery, diagnosis and treatment of cancer before metastasis through a test for exosomes could significantly improve the cure rate and survival rate of patients [10–13].

Through separating exosomes from blood and testing their types and characters, useful information may be acquired for the early prediction of cancer metastasis [14–18]. Separation of exosomes from similar-sized particles is challenging due to the complexity of biological fluids. The most common method used for isolating exosomes is ultracentrifugation (UC) [19,20]. A centrifugal force is applied to the sample to sediment the more dense molecules, such as intact cells and large debris, to form pellet. After the pellet is removed, the supernatant is subjected to an increased centrifugal force. Then, exosome purification may be done through repeated centrifugations. In ultracentrifugation, the applied centrifugal force might reach 200,000 × g. However, a combination of techniques is necessary to isolate a pure population of exosomes. Differential and density gradient ultracentrifugation based on size and density have been demonstrated to improve purity. Alternatively, a precipitation technology for exosome isolation has been developed by using polymer nets to capture exosomes that can be recovered by a low speed centrifugation [21]. This method traps EVs through a porous microstructure. In addition, the immuno-affinity purification (IP) approach captures specific exosomes by relying on the receptors on its surface [21]. The use of antibody-coated magnetic beads with the IP approach results in the high recovery and purity of exosomes. Filtration by sieving extracellular vesicles through a membrane is a straightforward approach, but the porous size of the membrane is an important consideration [19].

Exosomes are small, with diameters ranging from 30 to 100 nm. Even when the blood cells are removed, purification of exosomes from the serum still faces difficulty due to the existence of nanoscale proteins. For the precise testing of exosomes, it is necessary to remove the proteins in the serum to avoid interference. In view of the above, this study abandoned the traditional and inconvenient method of polymer precipitating and centrifugation to treat proteins. This study combined magnetic controlled nanoparticle technology [22,23] by coating the magnetic nanoparticles with polyethylene glycol and utilized them by controlling the motion of the nanoparticles to capture protein in the serum. The captured protein impurities were later separated and removed to the bottom of the beaker using a permanent magnet instead of a precipitator in the traditional method. The remained supernatant containing exosomes can be used for further analyses, such as cancer diagnosis. This could improve the efficiency of cancer detection by obtaining intact exosomes.

## Materials and methods

### Materials

The necessary chemicals needed to synthesize Fe_3_O_4_ MNPs include ferric chloride (FeCl_3_·6H_2_O), ferrous chloride tetrahydrate (FeCl_2_·4H_2_O), and sodium hydroxide (NaOH). They were purchased from the SHOWA Corporation (Japan). Polyethylene glycol (PEG), purchased from Alfa Aesar (USA), was coated on Fe_3_O_4_ MNPs.

Fetal bovine serum (FBS), which was adopted as the analyte for bioassays of the exosome purification, was purchased from Thermo Fisher Scientific (USA). Bicinchoninic acid (BCA) was also purchased from Thermo Fisher Scientific. In addition, CozyHi^™^ prestained protein ladder (PRL0202) was purchased from HighQu GmbH (Germany).

### Exosome purification method

**Fig 1:**
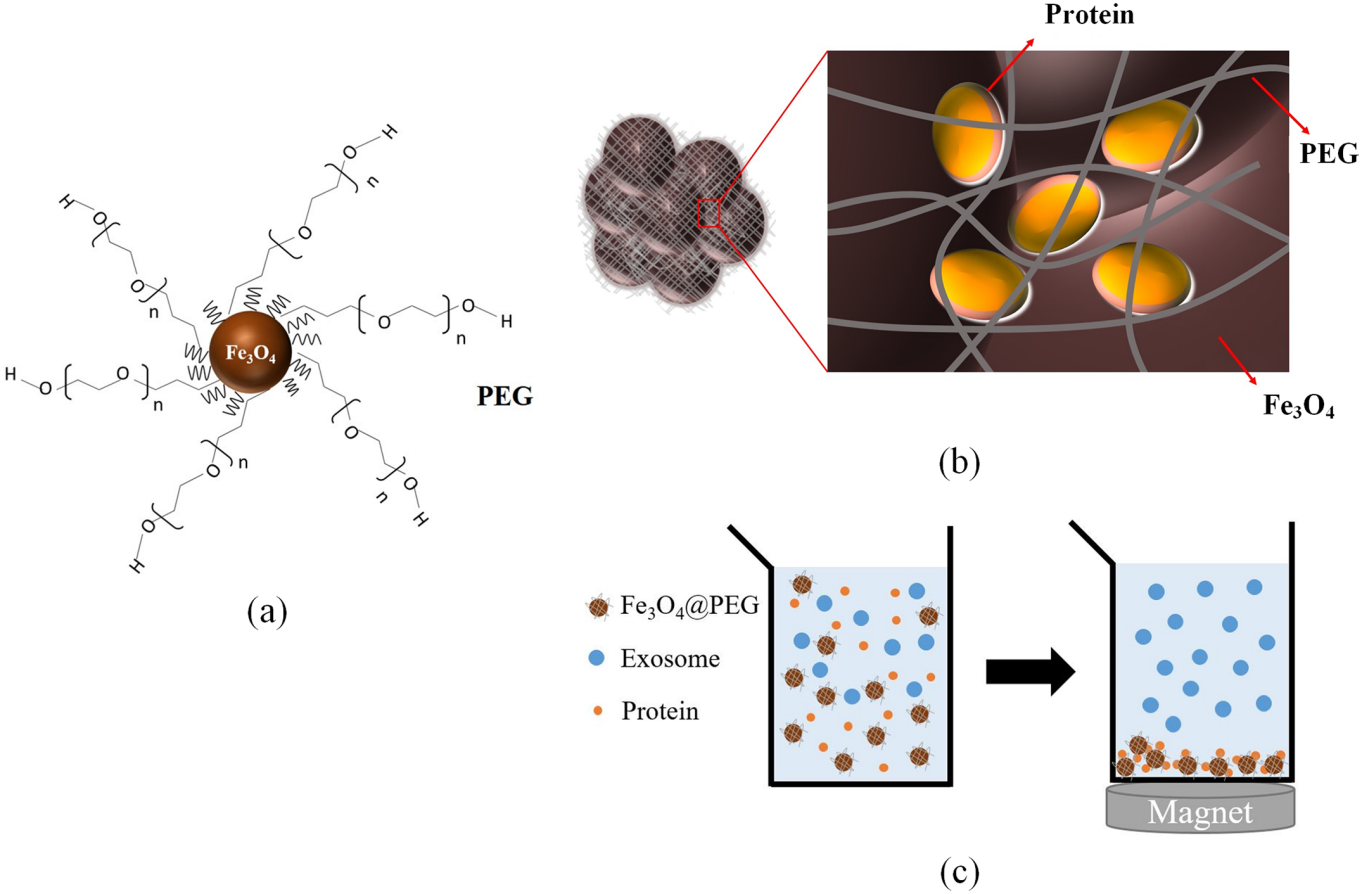
Exosome purification method using PEG-coated Fe_3_O_4_ MNPs: (a) branched PEG immobilized on MNP; (b) protein entrapped by the reticular structures of PEG; (c) removal of proteins using a permanent magnet.

Polyethylene glycol is a water-soluble polymer and its structure is commonly expressed as H–(O–CH_2_–CH_2_)_n_–OH. It is commonly used as a precipitant for protein crystallization in biological studies to gain the atomic structure of the proteins and to concentrate viruses in microbiology. The precipitation principle of proteins has not had conclusive proof. In addition, protein precipitation is not efficient. Instead of coating PEGs on a flat substrate, this study coated them on nanoparticles. Due to the large surface-to-volume ratio of nanoparticles, more branched PEGs can be immobilized on MNPs and the PEG chains can form reticular structures (Fig 1a). Moreover, MNPs are able to gather together to form agglomerates, resulting in a large number of holes. Proteins and tiny impurities can be entrapped in the holes of MNP agglomerates and the reticular structures of PEG (Fig 1b). Therefore, exosomes can be purified by removing the proteins using a permanent magnet (Fig 1c).

### Synthesis of PEG-coated Fe_3_O_4_ MNPs

Fe_3_O_4_ MNPs are biologically inert, have low toxicity, and have the advantage of superparamagnetic properties. The co-precipitation method is a common technique used to synthesize Fe_3_O_4_ MNPs via the reaction of iron (II) chloride tetrahydrate and iron (III) chloride hexahydrate with sodium hydroxide. The reaction is shown below:

$${\text{FeCl}}_{2}+2{\;\text{FeCl}}_{3}+8\;\text{NaOH}\to {\text{Fe}}_{3}{\text{O}}_{4}+8\;\text{NaCl}+4{\;\text{H}}_{2}\text{O}.$$ (1)

In order to prepare PEG-coated Fe_3_O_4_ MNPs, the co-precipitation reaction was modified. The first synthesis step was to mix 0.5 M of FeCl_2_·4H_2_O (8.9 g with 100 g of deionized (DI) water) solution with 1 M of FeCl_3_·6H_2_O (27 g with 100 g of DI water) solution in a beaker. The mixture was stirred and heated at 60°C, followed by adding PEG-6000 (41.629 g) such that the PEG had a content of 15 wt%. Then, stirring was continued for an additional 10 min. Next, 2.5 M of NaOH (15 g with 150 g of DI water) solution was slowly introduced into the mixture until the mixture had a pH of 11. After reacting for 30 min, the mixture was cooled to room temperature. The products were collected at the bottom of the beaker using a permanent magnet, and the supernatant was discarded. The nanoparticles were then rinsed with DI water using an ultrasonic shaker for 5 min. The washing process was repeated until the solution had a pH of 7. Finally, the nanoparticles were vacuum-dried at 50°C.

Several tests were then carried out to check the characterization of the PEG-coated Fe_3_O_4_ MNPs, including the topography, size distribution, molecular structure, phase composition and structure, and magnetic property.

### Exosome purification process

Prior to the exosome purification process, the protein concentration in the FBS must be quantified to determine the removal efficiency of the PEG-coated Fe_3_O_4_ MNPs. Thus, the procedure was started with diluting the FBS to half its original concentration, followed by adding BCA to react at 80°C for one hour. Then, a UV-VIS spectrophotometer SpectraMax 190 (Molecular Devices, LLC., USA) was employed to quantitatively measure the absorption spectrum under a light source with a wavelength of 560 nm. The amount of light absorbed is related to the protein content in FBS. The entire procedure was repeated several times by diluting the FBS to half its current concentration. Finally, a standard concentration curve of protein was established.

Next, the as-prepared PEG-coated Fe_3_O_4_ MNPs were poured into DI water to form a 0.6 wt% aqueous solution. In the meantime, the solution was shaken using an ultrasonic cleaner to uniformly disperse the MNPs. The solution (100 μL) was then mixed with FBS (100 μL) and thorough shaken for 30 min to allow the PEG-coated MNPs to capture protein in the serum. After the reaction, the MNPs were collected at the bottom of the beaker using a permanent magnet, and the supernatant was analyzed.

The proposed exosome purification method via PEG-coated Fe_3_O_4_ MNPs was then compared to the centrifugal method which is commonly used to isolate exosomes. Since FBS does not contain intact cells and large debris, the centrifugal process was conducted at 3000 rpm for 30 min in this study. A PEG aqueous solution was added to the FBS for centrifugation so that both methods would have the same PEG solution basis. To prevent damage to the exosomes during the centrifugal process, no precipitant was added. Moreover, the pH of the FBS was not adjusted. Three different PEG solution recipes were used: PEG-4000, PEG-6000, and PEG-8000. The concentrations of the PEG were set at 15% and 30% for comparison.

To study whether the increase of the MNP concentration would cause the promotion of protein removal, three different weight percentages of MNP in FBS were used: 0.6 wt%, 1.8 wt%, and 3 wt%. Each mixture was thoroughly shaken for 30 min., and the supernatant was then analyzed after the PEG-coated Fe_3_O_4_ MNPs were collected. In addition, the efficiency of the exosome purification was studied. Exosomes should not be removed by PEG-coated Fe_3_O_4_ MNPs. Thus, the quantities of exosomes before and after purification were compared using a NanoSight LM10. Sodium dodecyl sulfate polyacrylamide gel electrophoresis (SDS-PAGE) was also conducted to analyze the results of the exosome purification. The protein ladder could offer a standard for the accurate molecular weight determination of the expressed proteins.

## Results and discussion

### Characterization of Fe_3_O_4_ MNPs

#### Topography

**Fig 2:**
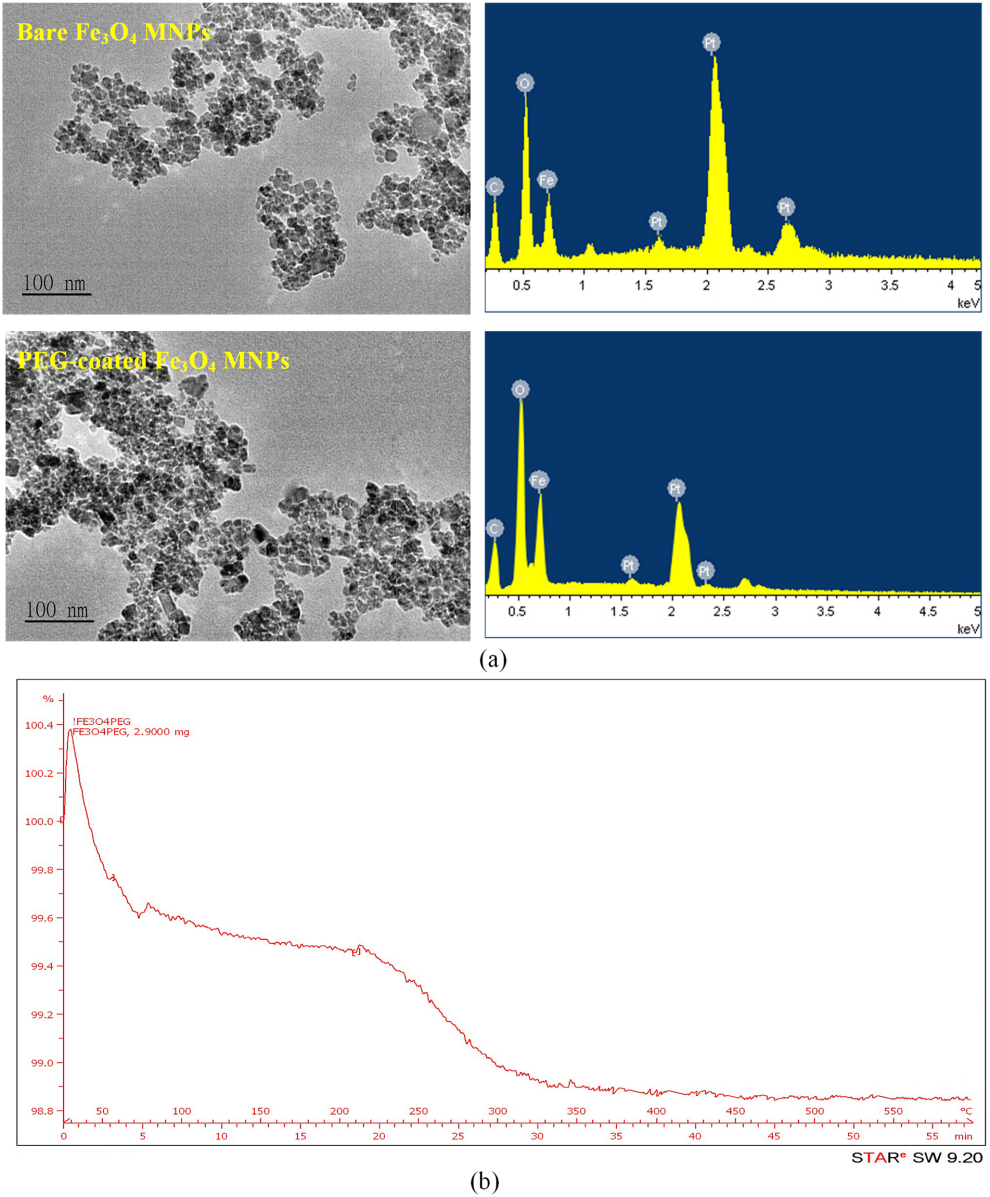
Characterization of Fe_3_O_4_ MNPs: (a) TEM images of Fe_3_O_4_ nanoparticles and their EDX analysis; (b) TGA analysis.

To observe the topography of the MNPs, a high resolution transmission electron microscope (TEM) (instrument model: JEM2010, JEOL Ltd., USA) was used. The TEM images of the MNPs are shown in Fig 2a. Obviously, the surface topography of the PEG-coated Fe_3_O_4_ MNP was similar to that of the bare Fe_3_O_4_ MNP. The analyses of the energy-dispersive X-ray spectroscopy (EDX) indicated that, for both materials, the chemical elements included Fe and O to form Fe_3_O_4_ MNPs. The element Pt came from the platinum coating for SEM scanning. Furthermore, thermogravimetric analysis (TGA) was conducted to determine the amount of PEGs included in MNPs. DuPont TA Q50 was used to continuously measure the mass of the sample as the temperature changed over time. Depending on the molecular mass, the flash point of PEG is approximately 200°C or higher. Therefore, the material caused thermal reaction incurring mass loss over the temperature range between 200 and 350°C was PEG. The TGA curve shows that the quantity of PEG was about 1% (Fig 2b).

#### Size distribution

**Fig 3:**
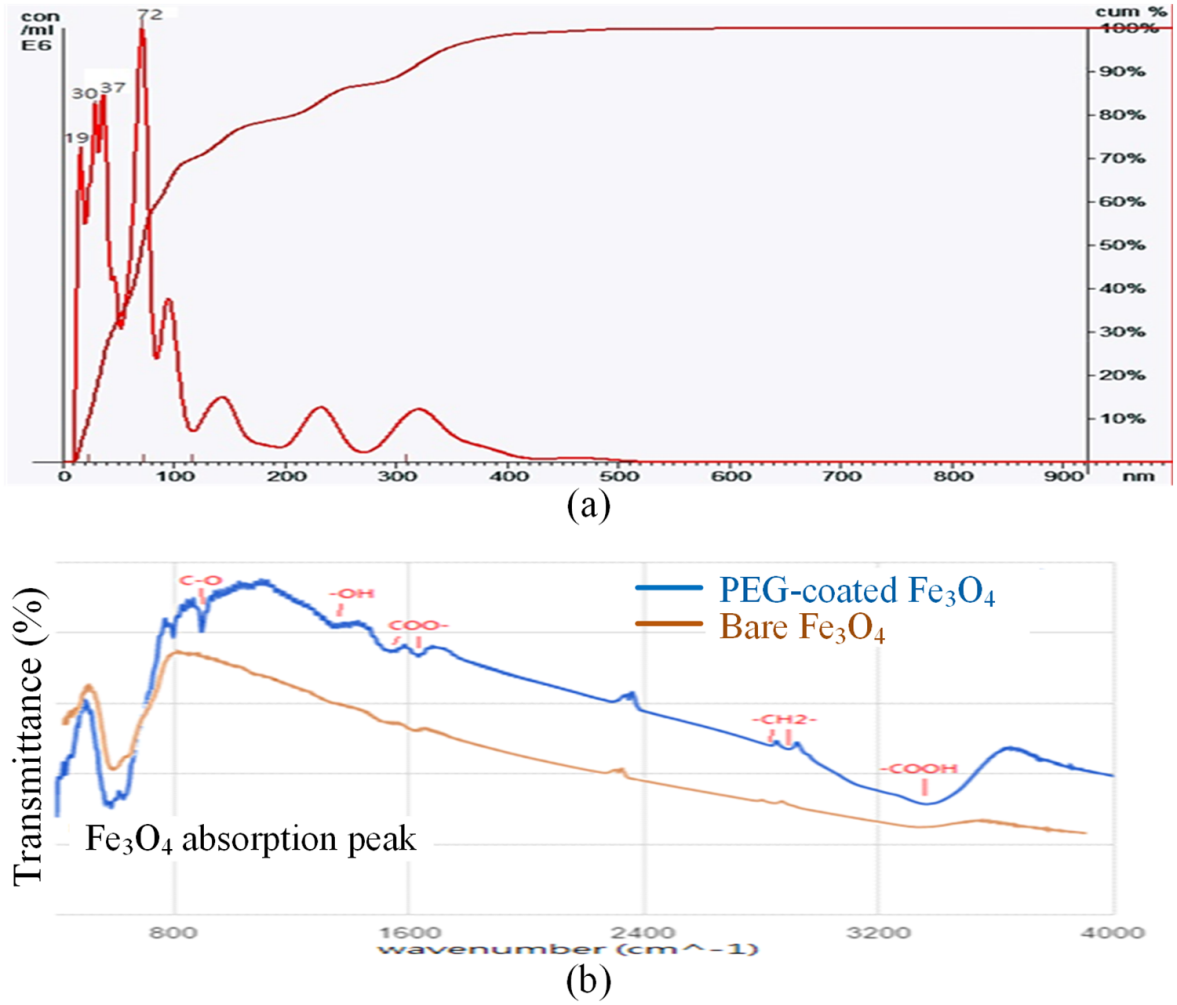
Characterization of Fe_3_O_4_ MNPs: (a) size distribution; (b) infrared spectra.

The size distribution of the PEG-coated Fe_3_O_4_ MNPs was analyzed using a NanoSight LM10 (Malvern Instruments Ltd, UK), which uses Nanoparticle Tracking Analysis (NTA) to obtain the size distribution and concentration measurements of particles in a liquid suspension. Fig 3a shows that the sizes of the PEG-coated Fe_3_O_4_ MNPs analyzed using NanoSight LM10 were distributed over a wide range. Some single MNPs existed with a size of 20 nm, while small agglomerates of MNPs measured about 30~37 nm in size. Large agglomerates coming from gatherings of small agglomerates had a size of 400 nm. Different particle sizes allowed PEG-coated Fe_3_O_4_ MNPs to capture various proteins.

#### Examination of functional groups

An FT/IR-4200 (JASCO International Co., Ltd., Japan), which is an instrument used for fourier transform infrared spectroscopy (FTIR), was used to determine the molecular structures of the PEG-coated Fe_3_O_4_ MNPs. As shown in Fig 3b, different molecular structures analyzed using FT/IR-4200 produced different infrared spectrum signals. An infrared absorption peak at 590 cm^-1^ was caused by Fe_3_O_4_, while a peak band at 3430 cm^-1^ was caused by the bonding between carboxyl on the surface of Fe_3_O_4_ and a hydrogen bond. This was evidence that PEG was coated on the Fe_3_O_4_ MNPs.

#### Crystallinity

**Fig 4:**
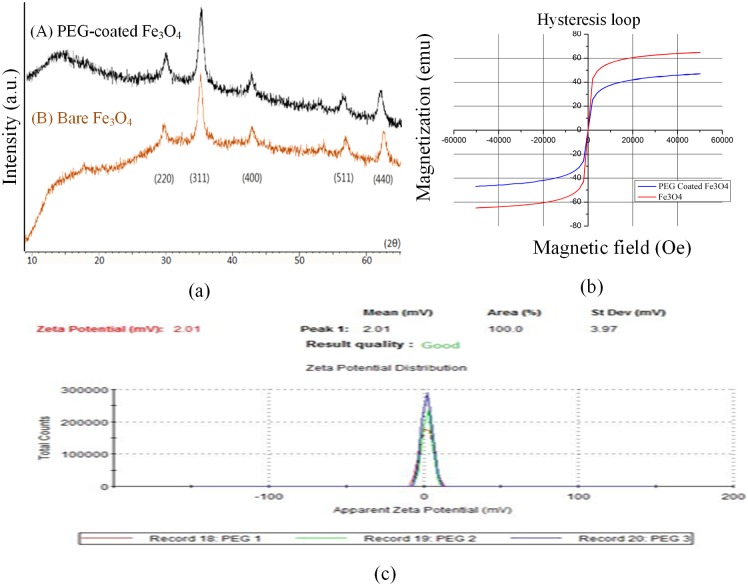
Characterization of Fe_3_O_4_ MNPs: (a) XRD of nanoparticles; (b) magnetic property of hysteresis; (c) zeta potential.

Detailed information about the phase composition and structure of the PEG-coated Fe_3_O_4_ MNPs was explored using X-ray diffraction (XRD). According to the analysis of XRD, the regular crystal of Fe_3_O_4_ had five characteristic peaks at angles (in degrees 2θ) of 30.1°, 35.4°, 43.1°, 56.9°, and 62.5°, respectively. These corresponded to the crystallographic lattices of (220), (311), (400), (511), and (440), respectively. As shown in Fig 4a, both the bare Fe_3_O_4_ MNPs and the PEG-coated Fe_3_O_4_ MNPs had characteristic peaks in their patterns that matched well with those of Fe_3_O_4_ (JCPDS No. 82–1533).

#### Magnetic property

A vibrating sample magnetometer (VSM) is an instrument used to measure the magnetic property of a sample. Under a sinusoidally vibrated magnetic field, the induced voltage in the sensing coils, due to changes in the magnetic flux, is proportional to the magnetic moment of the sample. Thus, this study employed an MPMS-7 SQUID VSM (Quantum Design, Inc., USA) to investigate the magnetic properties of the PEG-coated Fe_3_O_4_ MNPs. Since the experiments were carried out at an ambient temperature of 300°K, the magnetic susceptibility of the MNPs was measured under a magnetic intensity of ± 50,000 oersted (Oe). The measurement results using MPMS-7 SQUID VSM revealed that magnetic hysteresis did not occur for either the bare Fe_3_O_4_ MNPs or the PEG-coated Fe_3_O_4_ MNPs (Fig 4b). Moreover, the coercivity field could not be found from these two curves. These results agreed with those reported in [24] and demonstrated that the as-produced Fe_3_O_4_ MNPs had superparamagnetic properties. The saturation magnetization of the bare Fe_3_O_4_ MNPs reached 64.9 EMU/g, while only reaching 47 EMU/g for the PEG-coated Fe_3_O_4_ MNPs. Obviously, the magnetic polarization of MNPs was reduced by 27.6% due to the layer of PEG coating. However, the PEG-coated Fe_3_O_4_ MNPs kept their superparamagnetic properties, allowing them to be controlled by a permanent magnet.

#### Zeta potential

According to Coulomb’s law, the electrostatic force between opposite charges is attractive. The surface membrane of exosome is negatively charged and the zeta potential is around -18 mV. Our experimental result shows that the zeta potential of the PEG-coated Fe_3_O_4_ MNPs was 2.01 mV (Fig 4c). This zeta potential was too small to attract exosomes. That is, the PEG-coated Fe_3_O_4_ MNPs could not remove exosomes by attractive electrostatic force during the purification step.

### Removal of protein in FBS

#### Standard concentration curve of protein

To investigate the protein concentration curve, the FBS was diluted to half its current concentration repeatedly. The protein content in FBS was determined by measuring the absorption spectrum using a SpectraMax 190 UV-VIS spectrophotometer. A regression line representing the protein content was obtained as below:

y=0.132+0.004x (2)

**Fig 5:**
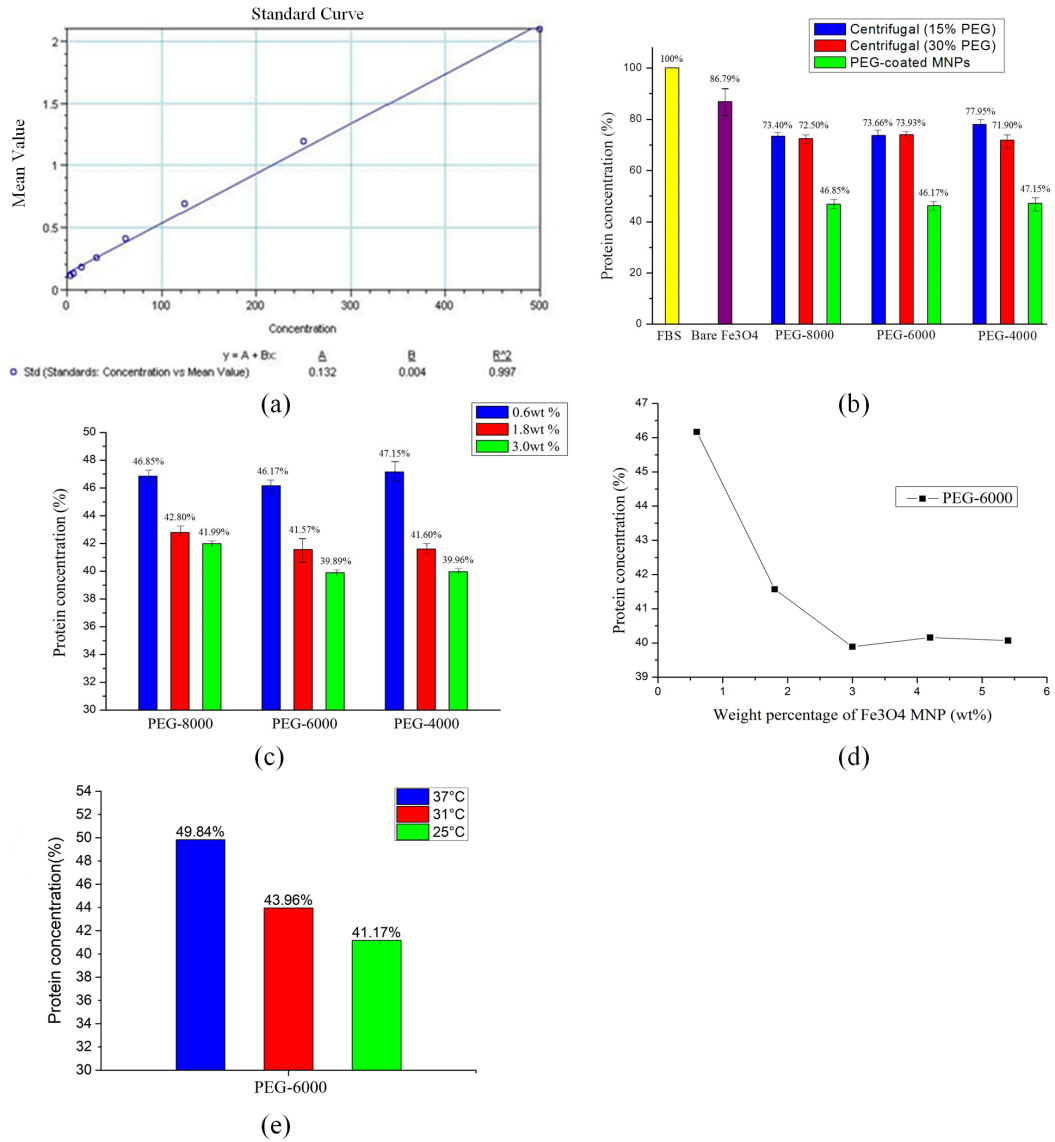
Protein removal efficiency of PEG-coated Fe_3_O_4_ MNPs: (a) standard concentration curve of protein in FBS; (b) comparison of removal methods; (c) influence of MNP concentration; (d) removal efficiency of PEG6000-coated MNPs at different weight percentages; (e) influence of temperature.

The result is shown in Fig 5a.

#### Comparison of removal methods

Fig 5b shows the comparison of protein removal between the proposed method and the centrifugal method. For centrifugal method, 30% PEG-4000 had the best protein removal effect, with the protein concentration dropping to 71.9% of the original solution. However, the Fe_3_O_4_ MNPs coated with PEG-6000 had extremely high efficiency. The protein concentration was reduced to 46.85%. However, the protein concentration dropped to 86.79% using the bare MNPs. Therefore, PEG-coated Fe_3_O_4_ MNPs had a higher efficiency than other methods to remove protein in FBS for exosome purification.

#### Influence of MNP concentration

The aforementioned protein removal procedure was conducted using three different weight percentages of MNP in FBS, i.e. 0.6 wt%, 1.8 wt%, and 3 wt%, to study the influence of MNP concentration on the effect of protein removal. As shown in Fig 5c, the 3 wt% MNPs coated with PEG-4000 and PEG-6000 had better protein removal efficiencies to reduce protein concentrations to 39.96% and 39.89%, respectively. However, it took several hours to collect the PEG4000-coated MNPs at the bottom of the beaker due to the suspension property of PEG-4000. It could not satisfy the time benefit, and thus, PEG-6000 was adopted for the follow-up experiments.

Next, Fe_3_O_4_ MNPs coated with PEG-6000 were added to the FBS to form five different weight percentages, from 0.6 wt% to 5.4 wt%. The experimental results for protein removal revealed that the removal efficiency increased as the weight percent of the MNPs increased. The protein removal efficiency reached a saturated state for a weight percent of MNPs greater than or equal to 3 wt% (Fig 5d). That is, the 3 wt% MNPs had the maximal removal efficiency. Increasing the MNP concentration did not promote protein removal. Furthermore, even when the MNPs were discarded after a protein removal procedure and, fresh PEG-coated Fe_3_O_4_ MNPs were added instead, the removal efficiency did not increase.

#### Influence of temperature

The influence of temperature on the efficiency of protein removal was studied using Fe_3_O_4_ MNPs coated with 3wt% PEG-6000. Three different temperatures were discussed: 37°C, 31°C, and 25°C (room temperature). As shown in Fig 5e, the protein concentration dropping to 41.17% of the original solution when the assay was conducted at 25°C. The efficiency of protein removal decreased with the rise in temperature. At 37°C, 50.16% of protein was removed, remaining 49.84% in the sample. Although most bio-related assays are conducted at 37°C, room temperature is adequate for protein removal to purify exosomes.

### Exosome purification assays

#### Amount of exosomes

**Fig 6:**
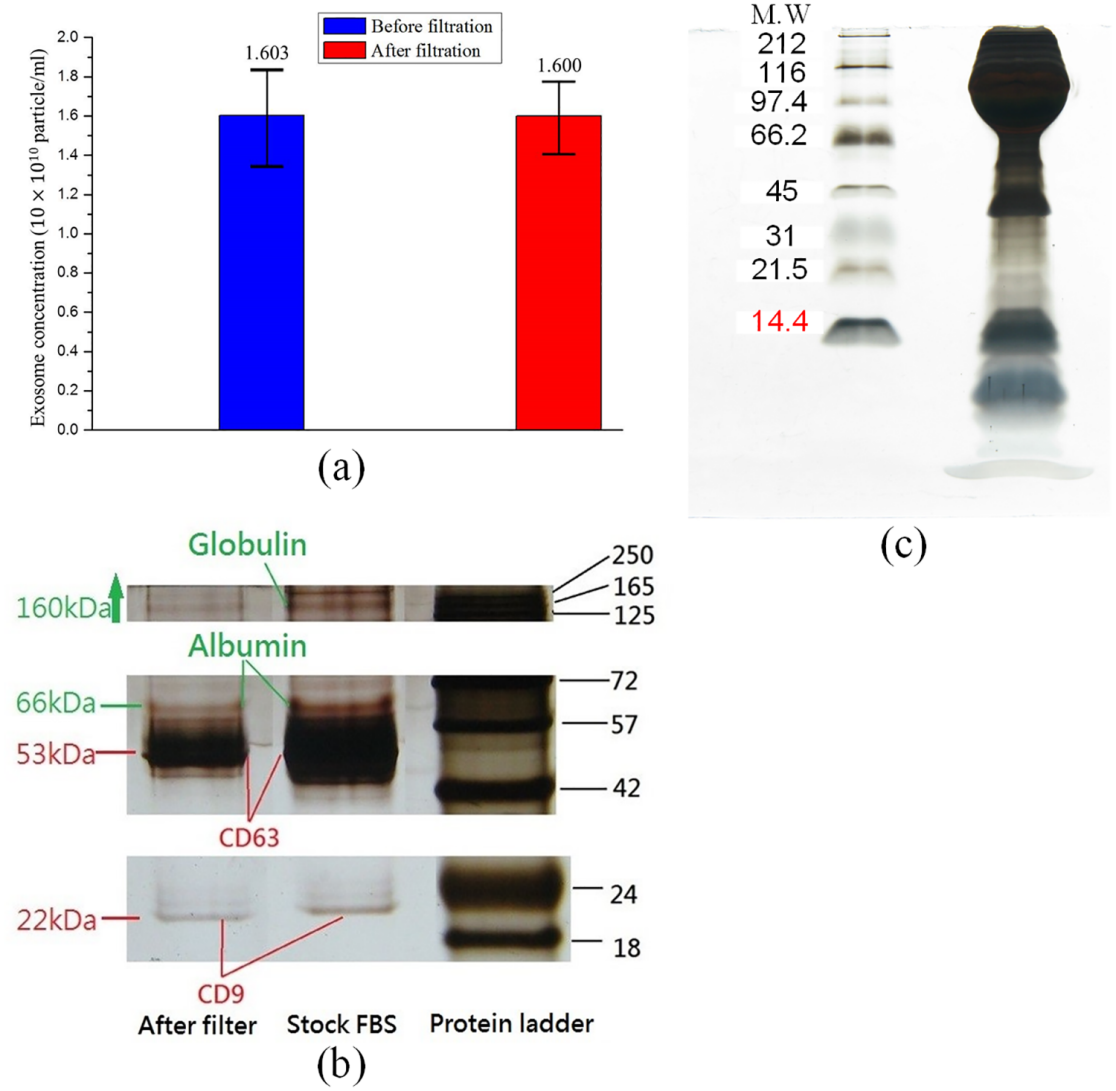
Exosome purification: (a) the quantities of exosomes before and after purification; (b) gel electrophoresis analyses of purified exosomes; (c) gel electrophoresis analyses of disrupted exosomes.

By the observation of the particle size distribution of 30~200 nm (the size range of various exosomes) using the NanoSight LM10, the exosome concentrations before and after purification were kept at about 1.6 × 10^10^ particles/mL (Fig 6a). Obviously, the exosomes were not affected by the PEG-coated Fe_3_O_4_ MNPs during the purification procedure.

#### Gel electrophoresis

After exosome purification, SDS-PAGE was conducted. The protein bands of the purified solution were compared with those of FBS (without protein removal), as shown in Fig 6b. The bands of CD63 (~53 kDa) and CD9 (~22 kDa) explained that exosomes existed in the FBS as well as in the purified solution. Both the blurred bands of the serum albumins (~66 kDa) and the various immunoglobulins (around 160 ~ 188 kDa) in the purified solution’s lane explained that most proteins in the FBS were removed by the PEG-coated Fe_3_O_4_ MNPs. The as-prepared MNPs were effective in purifying exosomes.

Next, SDS-PAGE was conducted again using the purified solution with the addition of 2-Mercaptoethanol (βME) to analyze the activity of exosomes. βME, a chemical compound with the formula HOCH_2_CH_2_SH, is commonly used to reduce disulfide bonds to disrupt the structure of proteins in biological studies. Hence, it was used in the gel electrophoresis to disrupt the structure of the exosomes by breaking the S-S bonds. As shown in Fig 6c, the disrupted exosomes released their contents so that nearly all the protein bands could not be discriminated. This experimental result explained that the PEG-coated Fe_3_O_4_ MNPs did not disrupt the exosomes. Obviously, the MNPs did not influence the activity of the exosomes.

#### Protein removal using human serum

The aforementioned procedure was applied to human serum for bioassays. Human serum was mixed with a 3 wt% solution of PEG6000-coated Fe_3_O_4_ MNPs and thoroughly shaken for 30 min. After reacting, the MNPs were collected at the bottom of the beaker using a permanent magnet, and the supernatant was examined. The protein concentration dropped to 50.57% of the original solution. This was better than the removal efficiency of the bare MNPs, which reduced the protein concentration to 86.32%. Nevertheless, the PEG6000-coated Fe_3_O_4_ MNPs were more effective in removing proteins in the FBS to 39.89% of the original solution. This study inferred that the coagulation factor fibrin in human serum, which is greater than that of albumin and globulin, was beyond the sizes of the holes in the MNP agglomerates or the reticular structures of PEG. Hence, they were not completely removed by the PEG-coated MNPs.

## Conclusion

Differential centrifugation is a commonly-used technique to isolate exosomes. The centrifugation steps include operations, for example, at 500 × g for 30 min and 2000 × g for 20 min at 4°C to eliminate cell debris, as reported by Kalra et al. [25]. It is time-consuming. However, the proposed approach of exosome purification using PEG-coated Fe_3_O_4_ MNPs is simple. By means of the reticular structures of PEG coated on Fe_3_O_4_ MNPs, proteins in FBS and human serum were successfully captured and precipitated using a magnet to accomplish the goal of exosome purification. The protein concentration in FBS was reduced to 39.89% of the original solution, without damaging the exosomes. Based the research results, the proposed technique could provide a solution to exosome purification and investigation.
